# Draft Whole-Genome Sequence of *Trichoderma gamsii* T6085, a Promising Biocontrol Agent of *Fusarium* Head Blight on Wheat

**DOI:** 10.1128/genomeA.01747-15

**Published:** 2016-02-18

**Authors:** Riccardo Baroncelli, Antonio Zapparata, Giulia Piaggeschi, Sabrina Sarrocco, Giovanni Vannacci

**Affiliations:** Dipartimento di Scienze Agrarie, Alimentari e Agro-Ambientali, Università di Pisa, Pisa, Italy

## Abstract

*Trichoderma gamsii* T6085 is a promising beneficial isolate whose effects consist of growth inhibition of the main agents causing *Fusarium* head blight, reduction of mycotoxin accumulation, competition for wheat debris, and reduction of the disease in both the lab and the field. Here, we present the first genome assembly of a *T. gamsii* isolate, providing a useful platform for the scientific community.

## GENOME ANNOUNCEMENT

*Fusarium* head blight (FHB) is one of the most important diseases of wheat, caused by a complex of *Fusarium* species, including *F. graminearum* and *F. culmorum*. The most serious consequence of FHB is the contamination of grain and cereal products with mycotoxins, with deoxynivalenol (DON) and its acetylated derivatives, 3- and 15-acetyl-DON, as the most frequently encountered trichothecene in FHB of wheat throughout Europe (1). Different strategies are used to reduce the impact of FHB, such as crop rotation, tillage practices, fungicide application, and resistant cultivars, but none of these by itself is able to reduce the impact of the disease. In this context, biological control offers, by the use of beneficial fungi, such as *Trichoderma* spp., a promising alternative for the management of FHB (2–4).

*Trichoderma gamsii* T6085 has been studied for many years by our research group for its ability to control FHB on wheat. From experimental data collected since then, T6085 resulted in being effective against *F. graminearum* and *F. culmorum* by inhibiting their growth (5), reducing mycotoxin contamination, particularly that of DON, and controlling the disease in both the lab and the field (6, 7). T6085 also was a good competitor against *F. graminearum* for cultural debris (8).

*T. gamsii* T6085 was sequenced using Illumina mate-paired sequencing technology by the Génome Québec Innovation Centre, part of McGill University (Canada). Mate-paired reads of 250 bp (3.80 Gbp total; average coverage, 100×) were assembled using Velvet 1.2.08 (9). The draft genome of *T. gamsii* consists of 381 scaffolds, with a total assembly length of 37.97 Mbp (*N*_50_, 417,961 bp; *N*_90_, 106,959 bp), 49.00% G+C content, and a maximum scaffold size of 1,198,811 bp. The completeness of the assembly was assessed using CEGMA version 2.4 (10), which estimated the genome sequence to be 97.58% complete based on full and partial targets. The nuclear genome was annotated using the MAKER2 pipeline (11). Overall, 10,944 protein-coding genes were predicted. Analysis with WolfPSORT (9) revealed that 1,356 predicted proteins (12.39% of the proteome) contain secretion signal peptides; those values are comparable to what has been reported for other *Trichoderma* genomes (12). A first comparative analysis within *Trichoderma* spp. (12, 13) and model organisms with publicly available genomes (*Fusarium* [14], *Neurospora* [15], *Colletotrichum* [16, 17], *Magnaporthe* [18], *Clonostachys* [19], and *Verticillium* (20)] suggested that *T. gamsii* T6085 contains a large number of specific carbohydrate-active enzymes (CAZy), such as GH30, GH2, GH13, and GH5, cerato-ulmin hydrophobin, proteinase inhibitor, peptidases, such as G1 and S53, and glucose/ribitol dehydrogenase enzymes. The genome sequence of *T. gamsii* T6085 represents a new resource that is useful for further research into the genetic bases of *Fusarium* head blight biological control by *Trichoderma* species.

### Nucleotide sequence accession numbers.

This whole-genome shotgun project has been deposited in GenBank under the accession no. JPDN00000000 (BioProject PRJNA252048). The version described in this paper is JPDN00000000.1.

## References

[1] Bottalico A, Perrone G 2002 Toxigenic *Fusarium* species and mycotoxins associated with head blight in small-grain cereals in Europe. Eur J Plant Pathol 108:611–624. doi:10.1023/A:1020635214971.

[2] Sarrocco S, Guidi L, Fambrini S, Degl’Innocenti E, Vannacci G 2009 Competition for cellulose exploitation between *Rhizoctonia solani* and two *Trichoderma* isolates in the decomposition of wheat straw. J Plant Pathol 91:331–338.

[3] Sarrocco S, Matarese F, Moretti A, Haidukowski M, Vannacci G 2012 DON on wheat crop residues: effects on mycobiota as a source of potential antagonists of *Fusarium culmorum*. Phytopathologia Mediterranea 51:225–235.

[4] Karlsson M, Durling MB, Choi J, Kosawang C, Lackner G, Tzelepis GD, Nygren K, Mukesh K, Dubey M, Kamou N, Levasseur A, Zapparata A, Wang J, Jensen B, Sarrocco S, Panteris E, Lagopodi AL, Pöggeler S, Vannacci G, Collinge DB 2015 Insights on the evolution of mycoparasitism from the genome of *Clonostachys rosea*. Genome Biol Evol 7:465–480 doi:10.1093/gbe/evu292. 25575496 PMC4350171

[5] Matarese F, Sarrocco S, Gruber S, Seidl-Seiboth V, Vannacci G 2012 Biocontrol of *Fusarium* head blight: interactions between *Trichoderma* and mycotoxigenic *Fusarium*. Microbiology 158:98–106. doi:10.1099/mic.0.052639-0. 21980117

[6] Perotto S, Angelini P, Bianciotto V, Bonfante P, Girlanda M, Kull T, Mello A, Pecoraro L, Perini C, Persiani AM, Saitta A, Sarrocco S, Vannacci G, Venanzoni R, Venturella G, Selosse MA 2013 Interactions of fungi with other organisms. Plant Biosyst 147:208–218. doi:10.1080/11263504.2012.753136.

[7] Sarrocco S, Matarese F, Moncini L, Pachetti G, Ritieni A, Moretti A, Vannacci G 2013 Biocontrol of *Fusarium* head blight by spike application of *Trichoderma gamsii*. J Plant Pathol S1:19–27.

[8] Sarrocco S, Baroncelli R, Zapparata A, Piaggeschi G, Valenti F, Vannacci G 2015 Beneficial fungi to improve food security: from the genome to the field on the highway of sustainability. J Food Process Technol 6:8. doi:10.4172/2157-7110.S1.024.

[9] Zerbino DR, Birney E 2008 Velvet: algorithms for *de novo* short read assembly using de Bruijn graphs. Genome Res 18:821–829. doi:10.1101/gr.074492.107. 18349386 PMC2336801

[10] Parra G, Bradnam K, Korf I 2007 CEGMA: a pipeline to accurately annotate core genes in eukaryotic genomes. Bioinformatics 23:1061–1067. doi:10.1093/bioinformatics/btm071. 17332020

[11] Cantarel BL, Korf I, Robb SM, Parra G, Ross E, Moore B, Holt C, Sánchez Alvarado A, Yandell M 2008 MAKER: an easy-to-use annotation pipeline designed for emerging model organism genomes. Genome Res 18:188–196. doi:10.1101/gr.6743907. 18025269 PMC2134774

[12] Baroncelli R, Piaggeschi G, Fiorini L, Bertolini E, Zapparata A, Pè ME, Sarrocco S, Vannacci G 2015 Draft whole-genome sequence of the biocontrol agent *Trichoderma harzianum* T6776. Genome Announc 3(3):e00647-15. doi:10.1128/genomeA.00647-15. 26067977 PMC4463541

[13] Kubicek CP, Herrera-Estrella A, Seidl-Seiboth V, Martinez DA, Druzhinina IS, Thon M, Zeilinger S, Casas-Flores S, Horwitz BA, Mukherjee PK, Mukherjee M, Kredics L, Alcaraz LD, Aerts A, Antal Z, Atanasova L, Cervantes-Badillo MG, Challacombe J, Chertkov O, McCluskey K, Coulpier F, Deshpande N, von Döhren H, Ebbole DJ, Esquivel-Naranjo EU, Fekete E, Flipphi M, Glaser F, Gómez-Rodríguez EY, Gruber S, Han C, Henrissat B, Hermosa R, Hernández-Oñate M, Karaffa L, Kosti I, Le Crom S, Lindquist E, Lucas S, Lübeck M, Lübeck PS, Margeot A, Metz B, Misra M, Nevalainen H, Omann M, Packer N, Perrone G, Uresti-Rivera EE, Salamov A, et al. 2011 Comparative genome sequence analysis underscores mycoparasitism as the ancestral life style of *Trichoderma*. Genome Biol 12:R40. doi:10.1186/gb-2011-12-4-r40. 21501500 PMC3218866

[14] Ma LJ, van der Does HC, Borkovich KA, Coleman JJ, Daboussi MJ, Di Pietro A, Dufresne M, Freitag M, Grabherr M, Henrissat B, Houterman PM, Kang S, Shim WB, Woloshuk C, Xie X, Xu JR, Antoniw J, Baker SE, Bluhm BH, Breakspear A, Brown DW, Butchko RA, Chapman S, Coulson R, Coutinho PM, Danchin EG, Diener A, Gale LR, Gardiner DM, Goff S, Hammond-Kosack KE, Hilburn K, Hua-Van A, Jonkers W, Kazan K, Kodira CD, Koehrsen M, Kumar L, Lee YH, Li L, Manners JM, Miranda-Saavedra D, Mukherjee M, Park G, Park J, Park SY, Proctor RH, Regev A, Ruiz-Roldan MC, Sain D, et al. 2010 Comparative genomics reveals mobile pathogenicity chromosomes in *Fusarium*. Nature 464:367–373. doi:10.1038/nature08850. 20237561 PMC3048781

[15] Galagan JE, Calvo SE, Borkovich KA, Selker EU, Read ND, Jaffe D, FitzHugh W, Ma LJ, Smirnov S, Purcell S, Rehman B, Elkins T, Engels R, Wang S, Nielsen CB, Butler J, Endrizzi M, Qui D, Ianakiev P, Bell-Pedersen D, Nelson MA, Werner-Washburne M, Selitrennikoff CP, Kinsey JA, Braun EL, Zelter A, Schulte U, Kothe GO, Jedd G, Mewes W, Staben C, Marcotte E, Greenberg D, Roy A, Foley K, Naylor J, Stange-Thomann N, Barrett R, Gnerre S, Kamal M, Kamvysselis M, Mauceli E, Bielke C, Rudd S, Frishman D, Krystofova S, Rasmussen C, Metzenberg RL, Perkins DD, Kroken S, et al. 2003 The genome sequence of the filamentous fungus *Neurospora crassa*. Nature 422:859–868. doi:10.1038/nature01554. 12712197

[16] O’Connell RJ, Thon MR, Hacquard S, Amyotte SG, Kleemann J, Torres MF, Damm U, Buiate EA, Epstein L, Alkan N, Altmüller J, Alvarado-Balderrama L, Bauser CA, Becker C, Birren BW, Chen Z, Choi J, Crouch JA, Duvick JP, Farman MA, Gan P, Heiman D, Henrissat B, Howard RJ, Kabbage M, Koch C, Kracher B, Kubo Y, Law AD, Lebrun MH, Lee YH, Miyara I, Moore N, Neumann U, Nordström K, Panaccione DG, Panstruga R, Place M, Proctor RH, Prusky D, Rech G, Reinhardt R, Rollins JA, Rounsley S, Schardl CL, Schwartz DC, Shenoy N, Shirasu K, Sikhakolli UR, Stüber K, et al. 2012 Lifestyle transitions in plant pathogenic *Colletotrichum* fungi deciphered by genome and transcriptome analyses. Nat Genet 44:1060–1065. doi:10.1038/ng.2372. 22885923 PMC9754331

[17] Baroncelli R, Sreenivasaprasad S, Sukno SA, Thon MR, Holub E 2014 Draft genome sequence of *Colletotrichum acutatum sensu lato* (*Colletotrichum fioriniae*). Genome Announc 2(2):e00112-14. doi:10.1128/genomeA.00112-14. 24723700 PMC3983289

[18] Dean RA, Talbot NJ, Ebbole DJ, Farman ML, Mitchell TK, Orbach MJ, Thon M, Kulkarni R, Xu JR, Pan H, Read ND, Lee YH, Carbone I, Brown D, Oh YY, Donofrio N, Jeong JS, Soanes DM, Djonovic S, Kolomiets E, Rehmeyer C, Li W, Harding M, Kim S, Lebrun MH, Bohnert H, Coughlan S, Butler J, Calvo S, Ma LJ, Nicol R, Purcell S, Nusbaum C, Galagan JE, Birren BW 2005 The genome sequence of the rice blast fungus *Magnaporthe grisea*. Nature 434:980–986. doi:10.1038/nature03449. 15846337

[19] Karlsson M, Durling MB, Choi J, Kosawang C, Lackner G, Tzelepis GD, Nygren K, Dubey MK, Kamou N, Levasseur A, Zapparata A, Wang J, Amby DB, Jensen B, Sarrocco S, Panteris E, Lagopodi AL, Pöggeler S, Vannacci G, Collinge DB, Hoffmeister D, Henrissat B, Lee YH, Jensen DF 2015 Insights on the evolution of mycoparasitism from the genome of *Clonostachys rosea*. Genome Biol Evol 7:465–480. doi:10.1093/gbe/evu292. 25575496 PMC4350171

[20] Klosterman SJ, Subbarao KV, Kang S, Veronese P, Gold SE, Thomma BP, Chen Z, Henrissat B, Lee YH, Park J, Garcia-Pedrajas MD, Barbara DJ, Anchieta A, de Jonge R, Santhanam P, Maruthachalam K, Atallah Z, Amyotte SG, Paz Z, Inderbitzin P, Hayes RJ, Heiman DI, Young S, Zeng Q, Engels R, Galagan J, Cuomo CA, Dobinson KF, Ma LJ 2011 Comparative genomics yields insights into niche adaptation of plant vascular wilt pathogens. PLoS Pathog. 7:e1002137. doi:10.1371/journal.ppat.1002137. 21829347 PMC3145793

